# Glass Refraction Distortion Object Detection via Abstract Features

**DOI:** 10.1155/2022/5456818

**Published:** 2022-03-24

**Authors:** Lei Cai, Chuang Chen, Qiankun Sun, Haojie Chai

**Affiliations:** ^1^School of Artificial Intelligence, Henan Institute of Science and Technology, Xinxiang 453003, China; ^2^School of Information Engineering, Henan Institute of Science and Technology, Xinxiang 453003, China; ^3^School of Electrical and Control Engineering, Shaanxi University of Science & Technology, Xi'an 710021, China

## Abstract

Glass reflection and refraction lead to missing and distorted object feature data, affecting the accuracy of object detection. In order to solve the above problems, this paper proposed a glass refraction distortion object detection via abstract features. The number of parameters of the algorithm is reduced by introducing skip connections and expansion modules with different expansion rates. The abstract feature information of the object is extracted by binary cross-entropy loss. Meanwhile, the abstract feature distance between the object domain and source domain is reduced by a loss function, which improves the accuracy of object detection under glass interference. To verify the effectiveness of the algorithm in this paper, the GRI dataset is produced and made public on GitHub. The algorithm of this paper is compared with the current state-of-the-art Deep Face, VGG Face, TBE-CNN, DA-GAN, PEN-3D, LMZMPM, and the average detection accuracy of our algorithm is 92.57% at the highest, and the number of parameters is only 5.13 M.

## 1. Introduction

With the widespread use of glass curtain walls in architectural design, it makes the building more beautiful and increases the lighting effect of the interior. At the same time, the use of dark glass also increases the difficulty of collecting interior information from the outside. In actual military operations, it is often necessary to collect and identify information on enemy personnel inside through glass walls or dark glass, but tinted glass will filter or add color information to the object. Different degrees of glass bending or uneven thicknesses make the refractive index of different regions of the glass change, causing distortion and deformation of the collected object with shifted position information [1]. At the same time, the presence of reflected images [2, 3] or stains on the glass surface will obscure the object information, resulting in missing or blurred information in parts of the acquired image, making it more difficult to achieve accurate identification of enemy objects under such circumstances.

In the process of collecting object information, in order to avoid the detection of enemy personnel, the algorithm is required to have good real-time, with fast search and object identification capabilities. Effective training of the model is an important prerequisite for detection algorithms, and the larger the number of samples and the stronger the training model, the better the detection effect [4]. But during the process of translucent glass detection, uncontrollable unknown factors such as uneven glass thickness or bending, scattering, reflection cause problems such as distortion and deformation, blurred or missing feature information that is difficult to predict in the collected sample images, making it difficult to label them accurately as well as to object training. Without accurate and sufficient training sample data, it is difficult to achieve effective training of the model, and it is even more impossible to guarantee the accuracy and robustness of the detection for striking objects.

**Figure 1:**
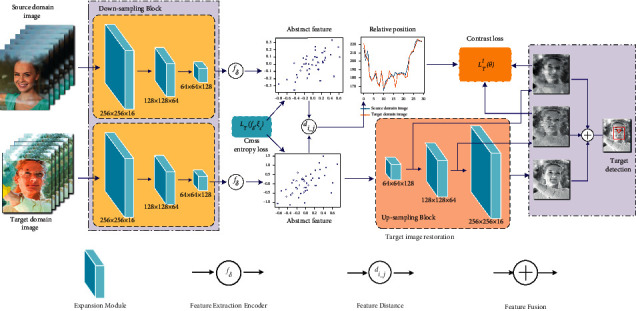
Flow chart of the detection method of object detection through the window.

In view of the above problems, this paper proposes a detection method for detecting objects through windows. Firstly, this paper improves the ResNet structure using skip connection and extension blocks to reduce the number of network parameters. Secondly, this paper extracts the abstract features of the object by binary cross-entropy loss. This feature ignores some details of the image and is highly distinguishable. Finally, the relative position relationship of each part feature is used to correct the distorted object image, which improves the accuracy of the algorithm for object detection. The specific process is shown in Figure 1.

In this paper, our main contributions include the following:

1. Improved deep residual neural network. The gradient disappearance problem is effectively reduced by introducing skip connections, and the expansion modules with different expansion rates are introduced to reduce the parameter training of the algorithm.
2. Propose a distortion object detection method. Combining the binary cross-entropy loss to abstractly extract the key feature information of the object, and using the relative distance relationship of the features to correct the image distortion caused by glass refraction, so as to increase the accuracy of the object identification and localization.
3. The algorithm in this paper was evaluated in the environment of glass refraction, glass reflection, and glass surface stain interference on the GRI dataset and achieved good performance.

## 2. Related Work

During through-window object detection, inconsistent refractive indices cause unpredictable distortions in the object image and random changes in the relative positional relationships of the object features [5]. The algorithm is difficult to repair the distorted and deformed images by means of refractive index inversion [6, 7] due to the inability to accurately capture the refractive index information of each part of the glass, which makes the detection process difficult. At the same time, the presence of stains or water mist on the glass surface and the use of tinted glass cause the captured images to be more blurred. Factors such as reflective contamination on the glass surface and object occlusion also make the identification process more difficult.

To address the glass reflection problem during detection, Zhang et al. [3] proposed to use an edge-aware cascade network to remove reflections, and thus reduce the effect of glass reflections when taking pictures through glass. To reduce the impact of glass reflections on the quality of acquired images, a multi-image-based depth estimation method using convolutional neural networks (CNN) was proposed in [2]. After first classifying the background and reflected images in the image, the reflected images are then removed, and finally, the removed background edges are regenerated using a generative adversarial network (GAN). Ref. [8, 9] proposed regularization methods based on wavelet transform and a network based on feature sharing strategy for the removal of reflective information from images, respectively.

In terms of reducing the effect of image distortion on object detection. Ref. [10] proposed a combination of different networks to restore the linear geometry of the face, thus reducing the effect of image distortion on detection. In the [11], an automatic correction method for omnidirectional image distortion based on a unified learning model (OIDC-Net) is proposed, which uses an attention mechanism with heterogeneous distortion coefficient estimation to achieve the correction of omnidirectional images. The above methods have excellent performance on the corresponding distorted images, but the above-distorted images have some regularity and can be adjusted by corresponding coefficients. For the distorted images caused by glass refraction, the irregularity of the glass and the unknown parameters make the distortion correction more difficult, and no corresponding research literature has been found.

With the rapid development of deep learning, the detection ability has been greatly improved, and the real-time performance of the algorithm is also highly required [12]. Qi et al. [13] used spatial relationships between local patches and facial features, which improved the accuracy and robustness of the algorithm for pose change and occlusion object tracking. Jian et al. [14] combine color contrast features of orientation features and background features to generate robust saliency maps, which makes the saliency detection model more effective and efficient. Cai et al. [15] propose an underwater distortion target recognition network. This method compensates for the problem of missing salient features by spatial semantic information, which effectively improves the accuracy of recognizing underwater distorted targets. Qian et al. [16] propose the Distortion Generation Network, which generates generous fish-eye images automatically using only small samples. Moreover, the Adversarial Distortion Generation Network is proposed to mine hard examples via adversarial training. These hard examples benefit detectors to be more robust to seriously distorted objects in fish-eye images.

In summary, there are some research in object detection and anti-interference. However, there are few pieces of research on object detection with through-window. Glass refraction and other factors cause distortion and blurring of the object, which still affects the accuracy of object detection and localization through the glass. In this paper, a skip connection and expansion module are added to the network to reduce the number of parameters of the algorithm. For the problems of distortion and feature blurring in the distortion images, the relative distance relationship of object features is corrected by domain contrast loss, which can increase the accuracy of object detection in the distortion images.

## 3. Proposed Method

### 3.1. Network Model

In order to reduce the number of parameters brought by the network and to improve the operational efficiency of the network, the optimized FSSNet [17] is used as the backbone for the distortion object detection network. Recoding the abstract features of the acquired translucent images using an encoder network to reduce the interference of factors such as color, brightness, and blur in the detection process. The abstract features are redecoded by the decoder combined with the feature relative position information to reduce the problem of position shift and image distortion caused by the inhomogeneous refractive index.

**Figure 2:**
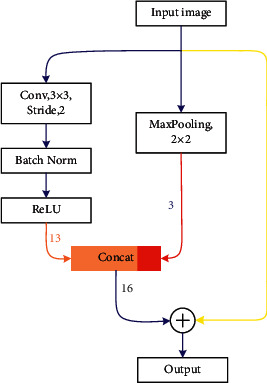
The base network module. The left side produces a 13-channel feature map (orange line in the figure), max pooling outputs a downsampled feature map with 3 channels (red line in the figure), and the yellow line in the figure indicates skip connections.

Let the input image be a 3-channel 256 × 256 image. The convolutional layer has 13 convolutional kernels. The convolution kernel size is 3 × 3 and the step size is 2. Convolution followed by batch normalization and rectifier linear unit (ReLU) for optimization, i.e., *Conv-BN-ReLU*. After processing, a feature map with 13 channels is obtained. An additional 2 × 2 maximum pooling operation is performed to obtain a 3-channel feature map. The two feature maps are fused to obtain a 16-channel feature map. At the same time, a skip connection is added to the input and output to enable the model to extract features better, which is shown in Figure 2.

**Figure 3:**
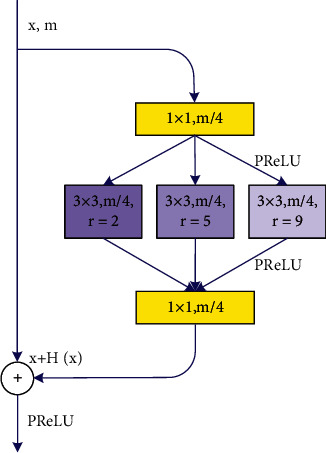
Expansion module.

To be able to extract feature information under different fields of view, the 3 × 3 convolution filter is set up as a convolution model with different dilation rates, i.e., a dilation module. The use of the dilation module helps the network to use fewer network layers to obtain more field of view, setting its dilation rate to 2, 5, and 9, respectively. The use of the dilation convolution expands the effective receptive field. As shown in Figure 3, *H* is usually defined as a set of convolutions followed by batch normalization and rectifier linear unit (ReLU), and the algorithm in this paper uses the parametric rectifier linear unit (PReLU) [18] as the activation function.

### 3.2. Distortion Image Correction Detection

#### 3.2.1. Abstract Feature Extraction (Encoder)

Currently, most algorithms are processed by feature extraction for each pixel of the original image, and the extracted features are used for subsequent object recognition or object detection. However, the human eye does not depend entirely on the information of each pixel of the image when detecting the process. For example, accurate detection can still be achieved when the human eye sees a sketch image or a distorted image of the object. In this paper, the feature extraction process is optimized based on this theory.

Let *S* and *T* denote the source and object domains, respectively, and the samples corresponding to *S* and *T* are denoted as {*x*_*s*_^*i*^}_*i*=1_^*N*^ and {*x*_*t*_^*i*^}_*i*=1_^*N*^, where *N* is the number of samples. The extraction of object features maps the sample *x* to a two-dimensional label space, which can be expressed as *ξ*_*S*_(*x*_*s*_^*i*^)⟶{0,1}, where *ξ*(·) is the mapping function.

Since the input image is distorted, not only do the object features need to be extracted in the feature extraction process, but also the relative position information of the features needs to be calculated and stored. The relative position information is obtained and then compared with the data information trained in the source domain, and the input image feature position information is corrected. So that the information in the extracted feature space is less affected by interference such as glass refraction and reflection. Let *f*_*δ*_ be the extracted feature encoder for the object abstract features. According to the binary cross-entropy loss, it is obtained that

(1) $$\begin{array}{c} \begin{array}{c} L_{T}\left(f_{\delta },\xi_{S}\right)=\frac{1}{N}\sum_{i}\left(-\xi_{S}\left(x_{s}^{i}\right)\log\left(f_{\delta }\left(x_{s}^{i}\right)\right)-\left(1-\xi_{S}\left(x_{s}^{i}\right)\log\left(1-f_{\delta }\left(x_{s}^{i}\right)\right)\right)\right) \end{array}, \end{array}$$

where *L*_*T*_(*f*_*δ*_, *ξ*_*S*_) is the empirical error of *f*_*δ*_ for the source domain labels. To improve the accuracy of the feature mapping function *ξ*(·), the following optimal mapping function *ξ*_*S*_^*∗*^(·) can be found by reducing the error:

(2) $$\begin{array}{c} \begin{array}{c} \xi_{S}^{*}\left(x_{s}^{i}\right)=\arg\underset{x_{s}^{i}}{\min}\left(L_{T}\left(f_{\delta },\xi_{S}\right)\right) \end{array}. \end{array}$$

**Figure 4:**
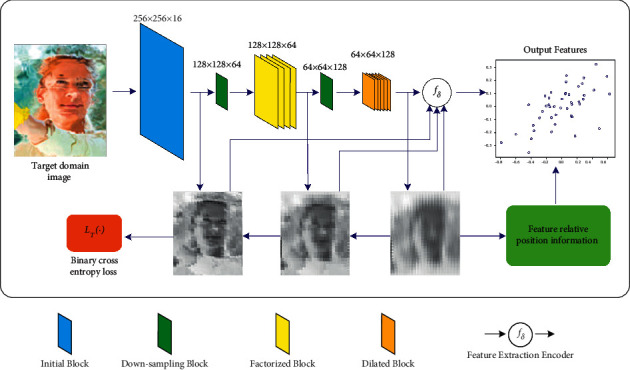
Abstract feature extraction process.

As shown in Figure 4, each feature of the input image is recorded in the 2D plane by the feature mapping function *ξ*(·), which makes the subsequent correction of distorted features simpler.

#### 3.2.2. Distorted Image Object Detection (Decoder)

The algorithm in this paper requires not only the extraction of features but also the determination of feature relative position relationships. Each feature of the input image is mapped in the 2D plane by the feature mapping function *ξ*(·), and the relative distance relationship *d*_*i*_*j*_ of the features is calculated, which can be expressed as follows:

(3) $$\begin{array}{c} d_{i_{j}}=\left|\delta_{i}\delta_{j}\right|,\delta_{i}\;\mathrm{and}\;\delta_{j}\in {\mathbb{R}}^{d}, \end{array}$$

where *d*_*i*_*j*_ is the distance between the feature point *δ*_*i*_ and the feature point *δ*_*j*_, and |·| is the distance calculation. The distance between features is stored at the same time during feature extraction, i.e., the relative position relationship of each feature is calculated.

Let *x*_*s*⟶*t*_^*i*^(*I*, *θ*) denote the learned training features, and construct a positive sample pair (*x*_*s*_^*i*^(*I*, *θ*), *x*_*s*⟶*t*_^*i*^(*I*, *θ*)) and (*x*_*s*_^*j*^(*I*, *θ*), *x*_*s*⟶*t*_^*j*^(*I*, *θ*)) for a batch of samples in the source domain *S* and a distorted image sample in the object domain *T*. The domain contrast loss from the source domain to the abstract feature space is as follows:

(4) $$\begin{array}{c} \begin{array}{c} L_{S}^{I}\left(\theta \right)=-\frac{1}{N}\sum_{i}\log\left(\frac{\exp\left(\text{sim}\left(x_{s⟶t}^{i}\left(I,\theta \right),x_{s}^{i}\left(I,\theta \right)\right)/\tau \right)}{\sum_{j}\exp\left(\text{sim}\left(x_{s⟶t}^{i}\left(I,\theta \right),x_{s}^{i}\left(I,\theta \right)\right)/\tau \right)}\right)-\frac{1}{N}\sum_{i}og\left(\frac{\exp\left(\text{sim}\left(x_{s}^{i}\left(I,\theta \right),x_{s⟶t}^{i}\left(I,\theta \right)\right)/\tau \right)}{\sum_{j}\exp\left(\text{sim}\left(x_{s}^{i}\left(I,\theta \right),x_{s⟶t}^{i}\left(I,\theta \right)\right)/\tau \right)}\right) \end{array}, \end{array}$$

where *x*_*s*_^*i*^(*I*, *θ*) is the feature from the last convolution layer of the sample image and *τ* is the temperature parameter. Similarly, the contrast loss from the object domain to the abstract feature space is as follows:

(5) $$\begin{array}{c} \begin{array}{c} L_{T}^{I}\left(\theta \right)=-\frac{1}{N}\sum_{i}\log\left(\frac{\exp\left(\text{sim}\left(x_{t}^{i}\left(I,\theta \right),x_{t⟶s}^{i}\left(I,\theta \right)\right)/\tau \right)}{\sum_{j}\exp\left(\text{sim}\left(x_{t}^{i}\left(I,\theta \right),x_{t⟶s}^{i}\left(I,\theta \right)\right)/\tau \right)}\right)-\frac{1}{N}\sum_{i}\log\left(\frac{\exp\left(\text{sim}\left(x_{t⟶s}^{i}\left(I,\theta \right),x_{t}^{i}\left(I,\theta \right)\right)/\tau \right)}{\sum_{j}\exp\left(\text{sim}\left(x_{t⟶s}^{i}\left(I,\theta \right),x_{t}^{i}\left(I,\theta \right)\right)/\tau \right)}\right) \end{array}. \end{array}$$

The autonomous correction of distorted images is achieved by reducing the contrast loss from the object domain to the abstract feature space; that is, the relative position relationship between the object domain and the abstract features in the source domain is reduced. The specific formulation is expressed as follows:

(6) $$\begin{array}{c} {\hat{L}}_{T}^{I}\left(\theta \right)=\arg\min L_{T}^{I}\left(\theta \right). \end{array}$$

At the same time, region-specific identification of the object *O* is achieved by using the location of the minima of the contrast between the source and object domains.

(7) $$\begin{array}{c} O=\arg\min\left(L_{S}^{I}\left(\theta \right)-{\hat{L}}_{T}^{I}\left(\theta \right)\right). \end{array}$$

**Figure 5:**
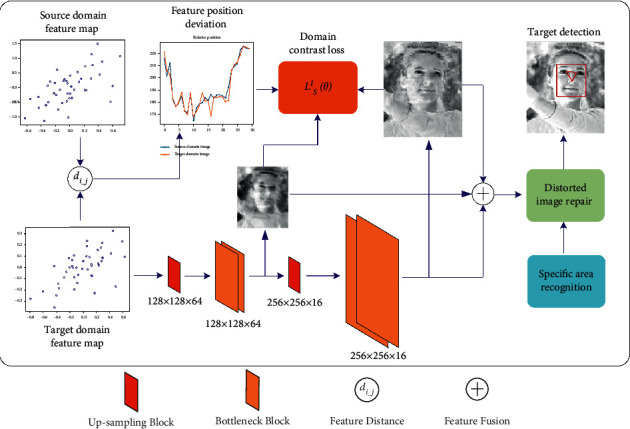
Distorted image reconstruction process.

When the distortion of the window acquisition image occurs, the image can be corrected and repaired according to the relative position relationship of the features, reducing the influence of the unknown refractive index information of the glass and the irregularity of the distorted shape on the image correction. In the decoding process, the abstract features are reduced and the relative positional relationships of the features are added at the same time. By adjusting the distance relationship of feature points, the repair of distorted and deformed images is realized, and the object feature position before refraction offset is accurately located, the specific process is shown in Figure 5.

### 3.3. Model Training

Due to the non-uniform refractive index of the window glass through which the acquisition process takes place and the specific data cannot be measured, the captured image undergoes unpredictable distortion. Therefore, the model is more suitable for training in an unsupervised manner. Settings *X*_*s*_ denote the source domain image information captured by the transmissive window and *Y*_*s*_ is the corresponding label. Assuming that *X*_*s*_ is the object domain acquisition image without labels, the feature information of the source domain image (*X*_*s*_, *Y*_*s*_) is extracted autonomously using the distortion object detection network, which can be expressed as follows:

(8) $$\begin{array}{c} \begin{array}{c} f_{\delta }\left(x\right)\in F,F\in {\mathbb{R}}^{\text{d}} \end{array}, \end{array}$$

where *x* ∈ *X*_*s*_ and ℱ is the feature space, and the distortion object detection network is trained to learn the encoder *f*_*δ*_(·) using a deep residual neural network.

In order to ensure that the network has good convergence and effective learning ability. Settings *x*_*i*_^*a*^ be an anchor sample, which can be calculated to obtain a positive sample *x*_*i*_^+^=argmax_*x*_*i*_^+^_*f*_*δ*_(*x*_*i*_^*a*^) − *f*_*δ*_(*x*_*i*_^+^)^2^, and also can be obtained a negative sample corresponding to *x*_*i*_^−^=argmax_*x*_*i*_^−^_*f*_*δ*_(*x*_*i*_^*a*^) − *f*_*δ*_(*x*_*i*_^−^)^2^.

The update process of the encoder *f*_*δ*_(·) can be written as follows:

(9) $$\begin{array}{c} \text{score}\left(f_{\delta }\left(x_{i}\right),f_{\delta }\left(x_{i}^{+}\right)\right)\gg \text{score}\left(f_{\delta }\left(x_{i}\right),f_{\delta }\left(x_{i}^{-}\right)\right), \end{array}$$

where *x*_*i*_ is the information of the *i*-th input, score(·) is a metric function to measure the similarity between samples, and the encoder *f*_*δ*_(·) is trained using the similarity information.

Using the vector inner product to calculate the similarity of two samples, the loss function *L*_*δ*_ can be expressed as follows:

(10) $$\begin{array}{c} L_{\delta }=-E_{X}\left[\log\frac{\exp\left(f{\left(x_{i}\right)}^{T}f\left(x_{i}^{+}\right)\right)}{\exp\left(f{\left(x_{i}\right)}^{T}f\left(x_{i}^{+}\right)\right)+\sum_{j=1}^{N-1}\exp\left(f{\left(x\right)}^{T}f\left(x_{i}^{-}\right)\right)}\right], \end{array}$$

where there are 1 positive sample and *N* − 1 negative samples in *x*_*i*_ samples. The purpose of learning is to make the features of *x*_*i*_ more similar to the features of the positive samples and less similar to the features of the *N* − 1 negative samples. This allows the abstract features extracted by the model to be more representative, enabling the algorithm to perform the task of window detection better.

## 4. Experimental Results and Analysis

### 4.1. Dataset and Evaluation Metrics

#### 4.1.1. Dataset

To verify the effectiveness of the proposed method, the homemade data Glass refraction image (GRI) dataset used in the simulation process, which is publicly available on GitHub (https://github.com/Robotics-Institute-HIST/Dataset/tree/master/Glass\%20\\Refraction\%20Image\%20dataset). There are 1267 original images with corresponding label files in this GRI dataset which is 1.60 GB. The images in the dataset contain a variety of interfering factors such as glass surface stains, water stains, reflections, refractions, and occlusions, which occur to varying degrees on the original image, and the dataset can be used to evaluate the performance of the distortion object detection algorithm.

#### 4.1.2. Implementation Details

In this paper, the optimizer uses stochastic gradient descent (SGD). The initial learning rate is 0.01. The momentum and weight decay are 0.9 and 0.0005, respectively. The initial learning rate is 0.01. The batch size is 64. The entire training process is iterated 60,000 times, where the learning rate decays with a decay rate of 0.1 when the number of iterations is 48,000 and 54,000. The initial values of the network satisfy a normal distribution with a mean of 0 and a variance of 2/*n*_*l*_.Where *n*_*l*_=*k*^2^*c*, *k* is the convolutional kernel size and *c* is the number of convolutional kernels.

#### 4.1.3. Evaluation Metrics

In this paper, accuracy, confidence, and IoU are used as the evaluation criteria of the distortion object detection algorithm.

(11) $$\begin{array}{c} \text{Accuracy}=\frac{T}{T+F}\times 100\%, \end{array}$$

where *T* is the number of faces correctly detected by the algorithm, *F* is the number of faces incorrectly detected by the algorithm, and Accuracy is the percentage of correct faces detected by the algorithm in the corresponding data set.

The confidence degree is the probability that indicates the detected object belongs to a certain category.

In order to accurately measure whether the algorithm can accurately detect the position of the real object, the IoU criterion is also used to measure the image detection results, and the deviation of the algorithm labeled position relative to the real position is determined by the magnitude of the IoU.

(12) $$\begin{array}{c} \begin{array}{c} \text{IoU}=\frac{O}{U} \end{array}, \end{array}$$

where *O* is an area of overlap and *U* area of union.

### 4.2. Analysis of Results

In this section, the effectiveness of the proposed algorithm for the distortion object detection task is verified by means of experimental comparative analysis. The algorithms in this paper are compared with existing excellent object detection algorithms, such as Deep Face [19], VGG Face [20], TBE-CNN [21], DA-GAN [22], PEN-3D [23], and LMZMPM [24]. The environment in which the algorithm runs: the CPU is Intel CORE i9 10900K and the graphics card is RTX 3090 VENTUS 3X 24 G.

#### 4.2.1. Simulation of the Algorithm in This Paper

**Figure 6:**
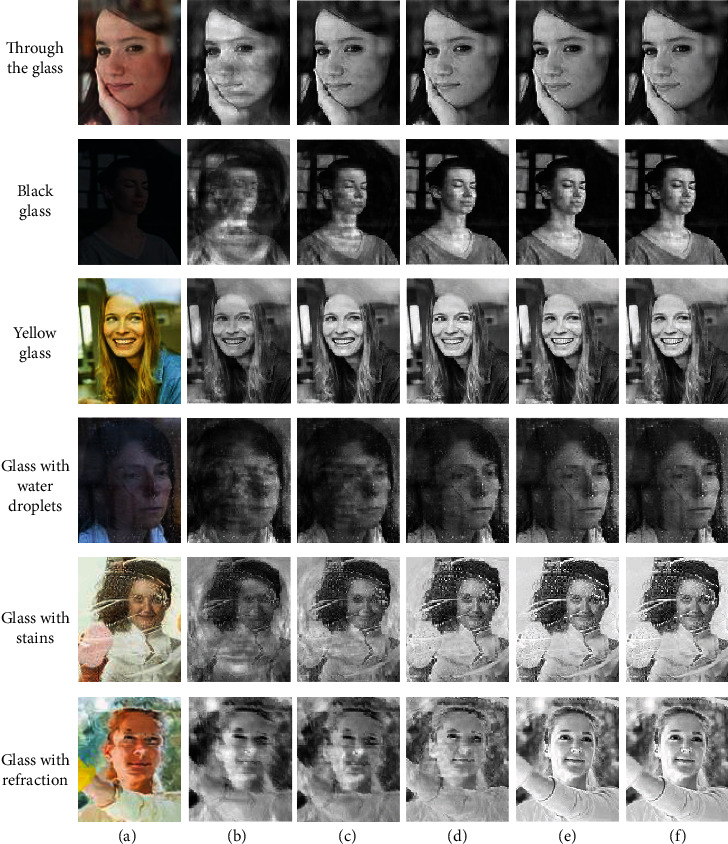
Simulation diagram of the processing process for the algorithm. From top to bottom: through the glass, black glass, yellow glass, glass with water droplets, glass with stains, and glass with refraction. (a) Original image, (b–e) convolution reconstruction of layer 1–4, and (f) output image.

As shown in Figure 6, there is the simulation of the image processing process for the algorithm. To increase the feature information of the generated image and reduce the influence of glass interference on object detection, the original image is reconstructed with only grayscale image output. In the first layer of the convolutional reconstruction result, the general outline information appears, but the reconstruction effect is not satisfactory, then the second, third, and fourth layers of the reconstruction process. The final output object image is not significantly different from the original image, and the object information is clearer. By comparing with the image in Figure 6, the reconstructed image reduces the effect of ordinary glass and tinted glass on the object image. In the reconstruction of the last row of glass refraction images, the algorithm in this paper corrects the key feature positions of the object. The distortion of object features caused by glass refraction is improved, and the output image is clearer than the original image without obvious distortion. Through the results of the study, we show that this paper's algorithm to process distortion images makes our processing effect more satisfactory.

**Figure 7:**
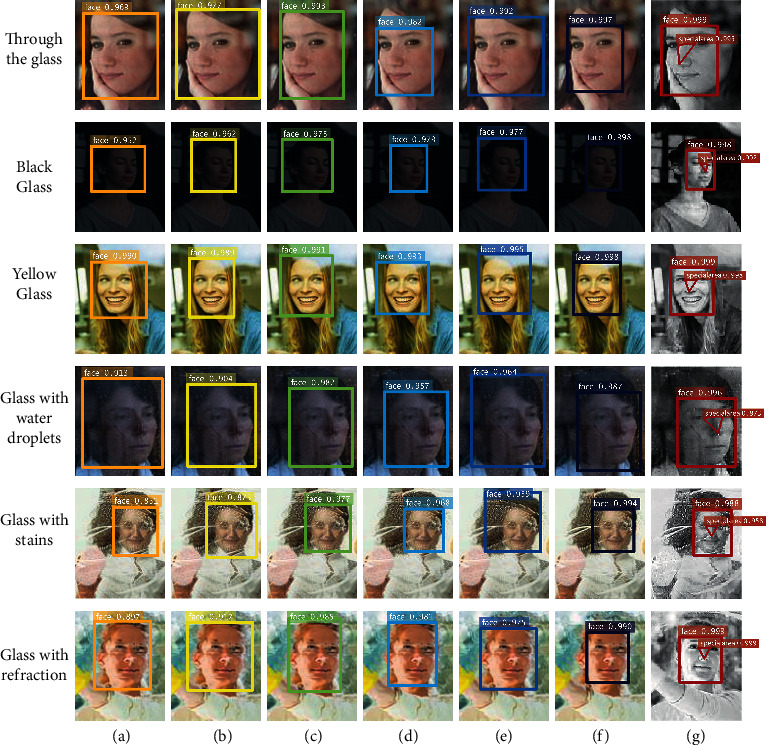
Detection results of different algorithms for distortion images. From top to bottom: through the glass, black glass, yellow glass, glass with water droplets, glass with stains, and glass with refraction. (a) Deep Face, (b) VGG Face, (c) TBE-CNN, (d) DA-GAN, (e) PEN-3D, (f) LMZMPM, and (g) Ours.

As shown in Figure 7, we test the detection ability of the algorithm in this paper, compared with Deep Face, VGG Face, TBE-CNN, DA-GAN, PEN-3D, and LMZMPM. The position of the detected object is marked in different colors and the detection label and confidence level are marked in the top left of the marking box. The detection confidence levels shown by each algorithm are relatively stable and have high values for the detection of transparent, black, and yellow glass images. The confidence values of each algorithm decreased when object detection was performed for water droplet interference, stain interference, and glass refraction images. Among the comparison algorithms, the Deep Face has the highest confidence level of 0.969 for the through-glass image, and the lowest confidence level of 0.851 for the glass stain interference image, with a maximum range of confidence variation of 0.118. The confidence level of the LMZMPM decreases from a maximum of 0.998 to a minimum of 0.987, with a minimum variation of 0.011. Among the other compared algorithms, VGG Face, TBE-CNN, DA-GAN, and PEN-3D confidence vary in the range of 0.102, 0.018, 0.036, and 0.036. The confidence level of the algorithm in this paper varies in the range of 0.011, where the highest confidence level is 0.999 for the glass-permeable image and the lowest confidence level is 0.988 for the stain-interference image. The confidence level of the algorithm in this paper is as low as 0.996 when removing images of the stain interference type, with a confidence variation range of only 0.003. At the same time, the algorithm in this paper marks special areas while detecting the object, which is beneficial to the application of subsequent military tasks.

#### 4.2.2. Detection Results on the GRI Dataset

**Figure 8:**
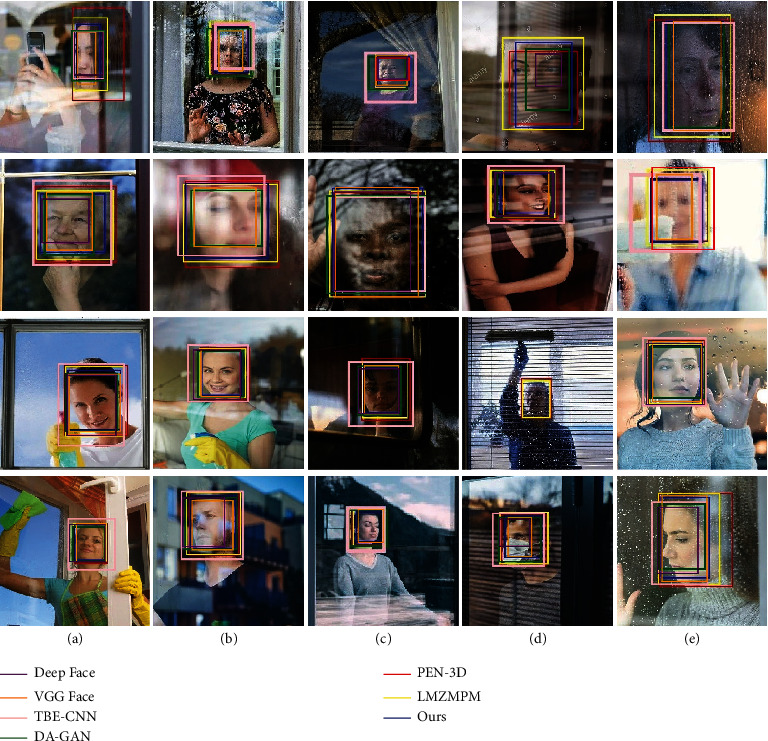
Glass reflection image detection results in GRI dataset. From left to right: (a) Normal, (b) bright light, (c) low light, (d) occlude, and (e) blurred.

**Figure 9:**
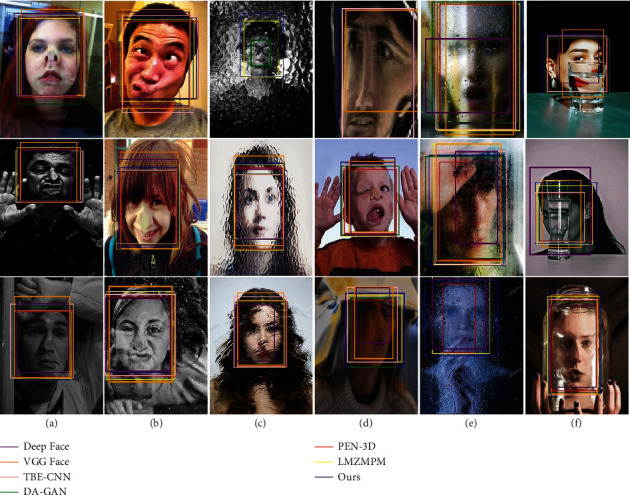
Glass refraction distortion results in the GRI dataset. From left to right: (a) Partially distorted, (b) facial distortion, (c) regular distortion, (d) irregular distortion, (e) color filter, and (f) occlude.

**Figure 10:**
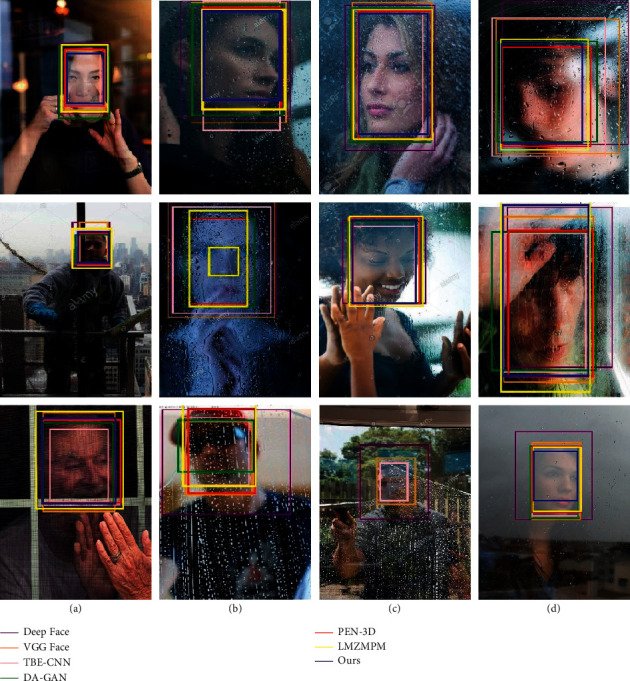
Results of detection of interference images on a glass surface in GRI dataset. From left to right: (a) Object occlusion, (b) water drop, (c) stains, and (d) blurry.

In order to verify the detection capability of the algorithm in distortion images, GRI datasets are created and classified into three types of images: glass reflection, glass refraction, and specular interference, which are shown from Figures 8to 10.

In the detection of the object through the window image, the effective information of the captured image is reduced due to the reflection of the glass, and this situation will have a significant impact on the accuracy of detection. The detection results of different algorithms are shown in Figure 8 and Table 1. In Figure 8, the images in each column show the reflected interference under different conditions, which include Normal, Bright light, Low light, Occlude, and Blurred. The LMZMPM has the highest detection accuracy rate of 95.85% with an IoU of 0.87 for the position prediction when detecting normal images. The detection accuracy of the proposed algorithm in this type achieves 95.57%, which is 0.28% lower than that of the LMZMPM, and the IoU of our algorithm in this paper is 0.92, which is 0.05 higher than the IoU of the LMZMPM. The DA-GAN and LMZMPM have the highest detection accuracy of 93.59% and 93.35% for bright light and occlude types. However, the algorithm achieved the highest detection accuracy and IoU for low light and blurred types, with a detection accuracy of 94.92% and 92.24, respectively, and IoU of 0.91 and 0.88, respectively. In the column of average, the highest detection accuracy and IoU of 93.88 and 0.88 are achieved by the algorithm in this paper. Although the detection result of this algorithm needs to be further improved under occlude type, our algorithm has the best overall performance. The above data proves the excellent detection performance and anti-interference ability of this algorithm for glass reflection images.

During the detection of distorted images caused by glass refraction, the object is detected, but deviations occur when predicting the position. For the above situation, the results of glass refraction interference image detection are compared and analyzed. In Figure 9, the refractive interference is classified into six interference cases: partially distorted, facial distortion, regular distortion, irregular distortion, color filter, and occlude. The results with the men are shown in Figure 9(b), the facial information of the object person is distorted by glass refraction. The Deep Face, PEN-3D, and LMZMPM detected the facial information uniformly under various conditions and do not consider the distortion effect caused by the glass on the object information. The results with the women are shown in Figure 9(d), the object's face is stretched laterally due to the effect of glass refraction. The Deep Face and the TBE-CNN detected the image information accurately, but there is a large error in the position prediction. The performance of the proposed algorithm is relatively stable under various interference conditions, and the error in position prediction is small, as shown in Table 2.

The detection results of glass refractive deformation images are shown in Table 2. We observe that the proposed algorithm simultaneously achieves the highest accuracy of 93.17% and IoU of 0.91 under the partially distorted condition. When dealing with facial distortion, our algorithm still achieves the highest detection rate, and the IoU performance is comparable to that of PEN-3D. Under color filter type and occlude type, LMZMPM performs best in accuracy rate, while the boundary boxes predicted by our method are more consistent with the ground truth, which is 0.83% and 0.21% higher than 92.62% and 92.83% of this paper's algorithm, respectively, but the IoU of this paper's algorithm are both the highest 0.82 and 0.80. In addition, our method has the best overall performance under regular distortion type and irregular distortion type. The proposed algorithm shows strong robustness and practicability when dealing with the five distortion types mentioned above.

In practical application scenarios, in addition to the interference of specular refraction and specular reflection, the mirror surface is likely to have stains or objects that introduce a lot of noise. In this experiment, we consider four cases: object occlusion, water droplets, stains, and glass blurring. The experimental results are shown in Figure 10. The results with the women shown in Figure 10(a), the bounding boxes predicted by the Deep Face and the VGG Face show large differences. From the data in Table 3, it can be obtained that the TBE-CNN labeled the most accurate object location when the object was obscured, with the maximum IoU of 0.90, but its detection accuracy was only 90.71%. And in this case, the detection accuracy of the algorithm in this paper is 94.31%, which is 3.6% higher than the detection accuracy of the TBE-CNN. When the glass is stained, the highest detection accuracy is 91.83% for the LMZMPM, which is 0.12% higher than our algorithm, but our algorithm got the highest IoU for 0.90. About the average, the algorithm in this paper has the highest detection accuracy of 92.68% and the highest IoU of 0.90.

**Figure 11:**
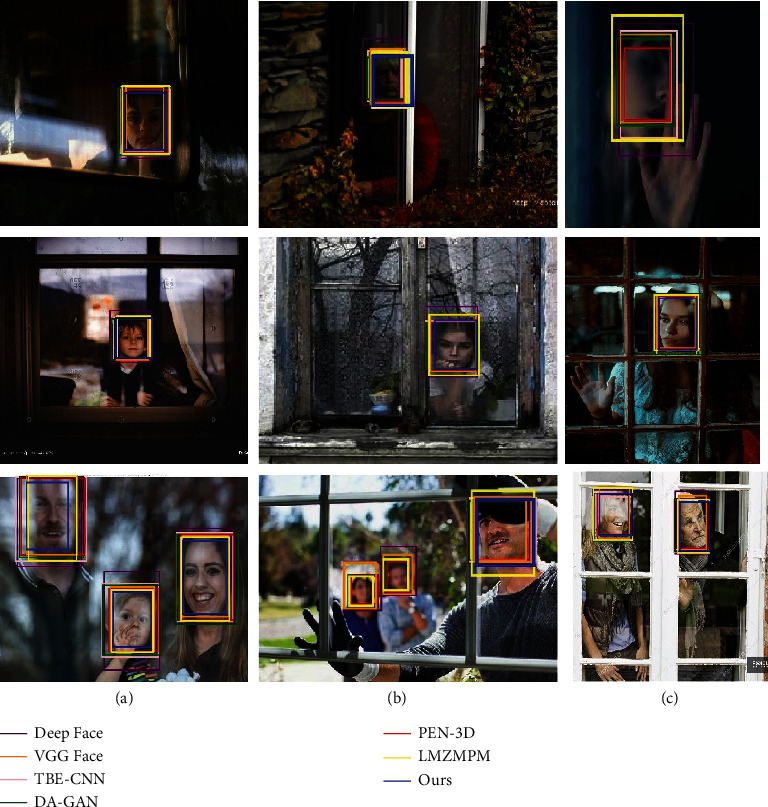
Long-range object detection results in GRI dataset. From top to bottom: dark environment, bright environment, multiple characters. (a) Environmental interference, (b) object occlusion, and (c) side face.

When detecting indoor objects through windows, in many cases the objects are at a distance, so it is more appropriate to simulate the objects at a long distance. In Figure 11, object detection is performed for ambient interference, object occlusion, and side-face conditions, while each type of image is subdivided into a dark environment, bright environment, and multi-person conditions. The specific detection accuracies of each algorithm are shown in Table 4.

The DA-GAN has the highest IoU of 0.91 in the case of environmental interference, but its detection accuracy is only 85.49%. Under the same condition, the highest detection accuracy of this paper's algorithm is 93.42%, and the IoU is 0.91, which is 0.01 lower than the IoU of the DA-GAN. In the case of object occlusion, the algorithm in this paper has the highest detection accuracy and IoU of 91.15% and 0.91, respectively. The highest detection accuracy of the LMZMPM is 94.77% in the detection of long-distance side face images, and the detection accuracy of the algorithm in this paper is 94.49%, which is 0.28% lower than that of the LMZMPM. But the IoU is 0.01 higher than the LMZMPM. Among the average data of the three cases, the detection accuracy and IoU of the algorithm in this paper are the highest at 93.02% and 0.91, respectively, which are 0.84% and 0.01 higher than the LMZMPM, and 8.15% and 0.03 higher than the DA-GAN.

The parameters and detection results of different algorithms are shown in Table 5. The average detection accuracy and IoU of the algorithm in this paper are the highest 92.57% and 0.88, respectively, the number of parameters of the algorithm is 5.13 M, and the FLOPs are 1.44 G. The number of parameters of PEN-3D is the smallest in these algorithms, which is 0.67 M smaller than ours, but its detection accuracy and IoU are only 84.06% and 0.81, and the FLOPs are 2.55 G, which is much lower than ours. The average detection accuracy and IoU of LMZMPM, which performs better for the data under a few interference conditions, are 91.16% and 0.85, respectively, which are slightly lower than the corresponding data of the algorithm in this paper. However, its number of parameters is 59.48 M, which is much larger than the number of parameters of the algorithm in this paper. The above data analysis shows that the algorithm in this paper has a smaller number of parameters and performs better when detecting through windows. Comparing Ours and Ours-1 reveals that reconstruction of distorted images can significantly improve the performance of the algorithm.

## 5. Conclusion

The simulation is validated by the glass reflection, glass refraction, and glass surface contaminant interference in the GRI dataset, and the detection under different types of light intensity, glass color, object occlusion, and image blurring are also considered. The data comparison analysis was performed by multiple simulations, and the average detection accuracy of the algorithm in this paper under different conditions was 92.57% with an IoU of 0.88, which was 1.41% and 0.03 higher than the LMZMPM and 4.57%, and 0.03 higher than the DA-GAN. The above data show that the algorithm has excellent detection ability and accurate position labeling ability when facing windowed images. However, there is a need to continue to improve the object detection of occluded images, so that the algorithm can show excellent detection and localization ability when facing different types of images.

## Figures and Tables

**Table 1:** Detection results of different algorithms in GRI dataset for glass reflection images (black font indicates the best data, underlined font indicates second best).

Algorithms	Normal		Bright light		Low light		Occlude		Blurred		Average	
	Acc(%)	IoU	Acc(%)	IoU	Acc(%)	IoU	Acc(%)	IoU	Acc(%)	IoU	Acc(%)	IoU
Deep face	85.82	0.82	81.27	0.75	82.95	0.77	86.02	0.83	83.42	0.79	83.90	0.79
VGG face	83.26	0.81	80.73	0.77	82.81	0.79	83.59	0.82	78.65	0.75	81.81	0.79
TBE-CNN	91.68	0.84	88.36	0.73	91.08	0.82	90.17	0.83	89.73	0.81	90.20	0.80
DA-GAN	92.71	0.90	**93.59**	0.86	91.96	0.87	92.24	**0.91**	90.79	0.85	92.26	0.86
PEN-3D	90.85	0.85	87.61	0.78	88.55	0.78	89.74	0.85	86.51	0.74	88.65	0.79
LMZMPM	**95.85**	0.87	92.71	0.85	92.15	0.82	**93.35**	0.87	91.17	0.80	93.05	0.83
Ours	95.57	**0.92**	93.38	**0.91**	**94.92**	**0.91**	93.31	0.90	**92.24**	**0.88**	**93.88**	**0.88**

**Table 2:** Glass refraction distortion image detection results of different algorithms in the GRI dataset (black font indicates the best data, underlined font indicates second best).

Algorithms	Partially distorted		Facial distortion		Regular distortion		Irregular distortion		Color filter		Occlude		Average	
	Acc(%)	IoU	Acc(%)	IoU	Acc(%)	IoU	Acc(%)	IoU	Acc(%)	IoU	Acc(%)	IoU	Acc(%)	IoU
Deep face	73.26	0.81	71.53	0.75	72.17	0.73	70.68	0.72	75.42	0.75	68.59	0.70	71.94	0.74
VGG face	77.65	0.72	76.15	0.71	74.52	0.75	72.43	0.74	78.35	0.76	71.61	0.72	75.12	0.73
TBE-CNN	81.28	0.78	79.63	0.76	78.35	0.77	75.61	0.73	80.53	0.79	77.16	0.76	78.76	0.77
DA-GAN	86.92	0.83	83.42	0.81	84.28	0.82	81.82	0.79	85.19	0.81	85.07	0.79	84.45	0.81
PEN-3D	84.47	0.71	82.61	**0.84**	81.26	0.71	80.56	0.70	83.07	0.73	82.65	0.75	82.44	0.74
LMZMPM	87.29	0.75	85.96	0.78	84.17	0.76	83.29	0.75	**93.45**	0.79	**93.04**	0.76	87.87	0.77
Ours	**93.17**	**0.91**	**91.08**	0.83	**90.15**	**0.83**	**84.31**	**0.81**	92.62	**0.82**	92.83	**0.80**	**90.69**	**0.83**

**Table 3:** Results of different algorithms for detection of glass surface interference images in GRI dataset (black font indicates the best data, underlined font indicates second best).

Algorithms	Target occlusion		Water drop		Stains		Blurry		Average	
	Acc(%)	IoU	Acc(%)	IoU	Acc(%)	IoU	Acc(%)	IoU	Acc(%)	IoU
Deep face	84.36	0.78	82.71	0.82	83.95	0.79	81.49	0.81	83.13	0.80
VGG face	88.54	0.79	85.62	0.75	81.37	0.73	82.05	0.78	84.40	0.76
TBE-CNN	90.71	**0.90**	87.38	0.86	85.29	0.82	83.72	0.85	86.78	0.86
DA-GAN	93.52	0.84	91.26	0.85	87.48	0.83	89.41	0.81	90.42	0.83
PEN-3D	86.74	0.88	85.29	**0.92**	82.74	0.87	83.18	0.86	84.49	0.88
LMZMPM	94.15	0.87	89.47	0.91	**91.83**	0.86	90.62	0.89	91.52	0.88
Ours	**94.31**	0.89	**92.42**	0.91	91.71	**0.90**	**92.27**	**0.91**	**92.68**	**0.90**

**Table 4:** Long-range object detection results of different algorithms on the GRI dataset (black font indicates the best data, underlined font indicates second best).

Algorithms	Environmental interference		Target occlusion		Side face		Average	
	Acc(%)	IoU	Acc(%)	IoU	Acc(%)	IoU	Acc(%)	IoU
Deep face	75.92	0.72	78.64	0.75	80.23	0.77	78.26	0.75
VGG face	78.54	0.74	81.52	0.76	83.57	0.75	81.21	0.75
TBE-CNN	80.71	0.78	79.38	0.81	82.34	0.79	80.81	0.79
DA-GAN	85.49	**0.91**	84.85	0.85	84.26	0.88	84.87	0.88
PEN-3D	79.85	0.85	80.17	0.82	81.95	0.85	80.66	0.84
LMZMPM	92.29	0.88	89.47	0.90	**94.77**	0.91	92.18	0.90
Ours	**93.42**	0.90	**91.15**	**0.91**	94.49	**0.92**	**93.02**	**0.91**

**Table 5:** A number of parameters of different algorithms and average detection results in multiple environments (black font indicates the best data, underlined font indicates second best, Ours-1 freezes distorted image reconstruction module).

Algorithms	FLOPs	Parameters	Average Acc	Average IoU
Deep face	2.7 G	114.44 M	79.31%	0.77
VGG face	1.6 G	131.94 M	80.64%	0.76
TBE-CNN	1.75 G	12.29 M	84.14%	0.81
DA-GAN	2.5 G	27.38 M	88.00%	0.85
PEN-3D	2.55 G	**4.46 M**	84.06%	0.81
LMZMPM	**0.72** G	59.48 M	91.16%	0.85

Ours	1.44 G	5.13 M	**92.57%**	**0.88**
Ours-1	—	—	85.24%	0.80

## Data Availability

The data used to support the findings of this study are available from the corresponding author upon request.

## References

[1] Brauers J., Schulte N., Aach T. (2008). Multispectral filter-wheel cameras: geometric distortion model and compensation algorithms. *IEEE Transactions on Image Processing*.

[2] Li T., Chan Y.-H., Lun D. P. K. (2021). Improved multiple-image-based reflection removal algorithm using deep neural networks. *IEEE Transactions on Image Processing*.

[3] Zhang H., Xu X., He H. (2020). Fast user-guided single image reflection removal via edge-aware cascaded networks. *IEEE Transactions on Multimedia*.

[4] Cai L., Sun Q., Xu T., Ma Y., Chen Z. (2020). Multi-AUV collaborative target recognition based on transfer-reinforcement learning. *IEEE Access*.

[5] Yazici B., Wang L. (2019). Analysis of artifacts in SAR imagery due to fluctuation in refractive index. *IEEE Transactions on Computational Imaging*.

[6] Papyan V., Elad M. (2016). Multi-scale patch-based image restoration. *IEEE Transactions on Image Processing*.

[7] Li S., Qin B., Xiao J., Liu Q., Wang Y., Liang D. (2020). Multi-channel and multi-model-based autoencoding prior for grayscale image restoration. *IEEE Transactions on Image Processing*.

[8] Huang Y., Quan Y., Xu Y., Xu R., Ji H. (2020). Removing reflection from a single image with ghosting effect. *IEEE Transactions on Computational Imaging*.

[9] Wan R., Shi B., Li H., Duan L.-Y., Tan A.-H., Kot A. C. (2020). CoRRN: cooperative reflection removal network. *IEEE Transactions on Pattern Analysis and Machine Intelligence*.

[10] Li Y.-H., Lo I.-C., Chen H. H. (2021). Deep face rectification for 360° dual-fisheye cameras. *IEEE Transactions on Image Processing*.

[11] Liao K., Lin C., Zhao Y., Gabbouj M., Zheng Y. (2020). OIDC-net: omnidirectional image distortion correction via coarse-to-fine region attention. *IEEE Journal of Selected Topics in Signal Processing*.

[12] Sun Q., Liu X., Bourennane S., Liu B. (2021). Multiscale denoising autoencoder for improvement of target detection. *International Journal of Remote Sensing*.

[13] Qi Y., Zhang S., Jiang F., Zhou H., Tao D., Li X. (2020). Siamese local and global networks for robust face tracking. *IEEE Transactions on Image Processing*.

[14] Jian M., Wang J., Yu H. (2020). Visual saliency detection by integrating spatial position prior of object with background cues. *Expert Systems with Applications*.

[15] Cai L., Chen C., Chai H. (2021). Underwater distortion target recognition network (UDTRNet) via enhanced image features. *Computational Intelligence and Neuroscience*.

[16] Qian Y., Yang M., Li H., Wang C., Wang B. (2021). Adversarial training-based hard example mining for pedestrian detection in fish-eye images. *IEEE Transactions on Intelligent Transportation Systems*.

[17] Zhang X., Chen Z., Wu Q. M. J., Cai L., Lu D., Li X. (2019). Fast semantic segmentation for scene perception. *IEEE Transactions on Industrial Informatics*.

[18] He K., Zhang X., Ren S., Sun J. Delving deep into rectifiers: surpassing human-level performance on imagenet classification.

[19] Taigman Y., Yang M., Ranzato M., Wolf L. (2014). Deepface: closing the gap to human-level performance in face verification. *Proc. IEEE Conf. Comput. Vis. Pattern Recognit.*.

[20] Schroff F., Kalenichenko D., Philbin J. FaceNet: a unified embedding for face recognition and clustering.

[21] Ding C., Tao D. (2018). Trunk-branch ensemble convolutional neural networks for video-based face recognition. *IEEE Transactions on Pattern Analysis and Machine Intelligence*.

[22] Zhao J., Xiong L., Li J., Xing J., Yan S., Feng J. (2019). 3D-Aided dual-agent GANs for unconstrained face recognition. *IEEE Transactions on Pattern Analysis and Machine Intelligence*.

[23] Liu F., Zhao Q., Liu X., Zeng D. (2020). Joint face alignment and 3D face reconstruction with application to face recognition. *IEEE Transactions on Pattern Analysis and Machine Intelligence*.

[24] Kar A., Pramanik S., Chakraborty A., Bhattacharjee D., Ho E. S. L., Shum H. P. H. (2021). LMZMPM: local modified zernike moment per-unit mass for robust human face recognition. *IEEE Transactions on Information Forensics and Security*.

